# Strategies for Individual Phenotyping of Linoleic and Arachidonic Acid Metabolism Using an Oral Glucose Tolerance Test

**DOI:** 10.1371/journal.pone.0119856

**Published:** 2015-03-18

**Authors:** Edoardo Saccenti, John van Duynhoven, Doris M. Jacobs, Age K. Smilde, Huub C. J. Hoefsloot

**Affiliations:** 1 Laboratory of Systems and Synthetic Biology, University of Wageningen and Research Center, Wageningen, The Netherlands; 2 Biosystem Data Analysis Group, University of Amsterdam, Amsterdam, The Netherlands; 3 Unilever Research & Development, Vlaardingen, The Netherlands; 4 Laboratory of Biophysics, University of Wageningen and Research Center, Wageningen, The Netherlands; 5 Netherlands Metabolomics Centre, Leiden, The Netherlands; Univeristy of California Riverside, UNITED STATES

## Abstract

The ability to restore homeostasis upon environmental challenges has been proposed as a measure for health. Metabolic profiling of plasma samples during the challenge response phase should offer a profound view on the flexibility of a phenotype to cope with daily stressors. Current data modeling approaches, however, struggle to extract biological descriptors from time-resolved metabolite profiles that are able to discriminate between different phenotypes. Thus, for the case of oxylipin responses in plasma upon an oral glucose tolerance test we developed a modeling approach that incorporates a priori biological pathway knowledge. The degradation pathways of arachidonic and linoleic acids were modeled using a regression model based on a pseudo-steady-state approximated model, resulting in a parameter A that summarizes the relative enzymatic activity in these pathways. Analysis of the phenotypic parameters *As* suggests that different phenotypes can be discriminated according to preferred relative activity of the arachidonic and linoleic pathway. Correlation analysis shows that there is little or no competition between the arachidonic and linoleic acid pathways, although they share the same enzymes.

## Introduction

A healthy human body is able to continuously adapt to its environment by rebalancing metabolic processes. This allows the body to restore homeostasis upon disturbances by daily stressors, such as diet and exercise. There is a growing awareness that the flexibility of the metabolic phenotype is a prerequisite for maintaining a healthy state [1] and metabolic phenotyping is receiving a great deal of attention [2,3,4,5].

The concept of metabolic resilience has also been adopted in a newly proposed definition for health, which departs from the ability to cope and adapt to social, physical and emotional challenges [6]. Loss of metabolic flexibility due to chronic stress, such as an unbalanced diet, has been associated with the onset of metabolic diseases, of which diabetes-2 is a pronounced example. Multiple molecular mechanisms, interconnected by complex regulatory networks, form the basis of metabolic flexibility.

At present there is only limited insight into whether and how phenotypes can differ in compensatory mechanisms to adapt to external stressors. There are also no quantitative phenotypical descriptors of metabolic flexibility. Recognition of phenotypes that differ in metabolic flexibility calls for a systems approach that addresses both the multifactorial aspects and the dynamic nature of metabolic regulatory processes upon external stressors. Advances in metabolic profiling now allow for broad coverage of the human metabolome. In combination with time-resolved sampling during the response phase this should provide a detailed view on the flexibility of the metabolic phenotype. Current data modeling techniques, however, struggle to extract quantitative phenotypical parameters from time-resolved metabolic profiles [7]. Modeling approaches to date do not take into account the fact that metabolites share pathways and that the time responses upon a dietary challenge are determined by consecutive catabolic or anabolic conversions [7].

We hypothesized that by incorporating such a priori biological knowledge, data modeling can be guided to deliver phenotypical descriptors that bear direct biological relevance. Furthermore, we expect that such an approach can also cope with a limited number of volunteers and sampling points [8].

In a recent study we observed significant inter-individual differences in the response of the different oxylipins upon oral glucose tolerance test [9] and thus we selected this study to derive quantitative descriptors of phenotypical pathway preferences. This was accomplished by building a regression model that incorporates both the time-resolved oxylipin concentrations and the a priori knowledge on the degradation pathways of arachidonic and linoleic acid. We show that the resulting parameters are adequate estimates of phenotypic descriptors of oxylipin metabolism.

## Overview of Oxylipins Metabolism

Oxylipins, in general, have been associated with the initiation and resolution of inflammation and vascular function [10] and are of particular interest for our purpose, since they follow specific pathways depending on the activity of different enzymes.

A wealth of prior information on conversion pathways of oxylipins is already available [11]. Here we will focus on those oxylipins that are enzymatically generated by oxidation of arachidonic acid and linoleic acid [12,13].

Arachidonic acid (AA) is a polyunsaturated fatty acid (PUFA) formed by the 6Δ/5Δ desaturase pathway from LA [14]. AA is metabolized by three enzymes: cyclooxygenase (COX)[15], lipoxygenase (LOX) [16], and cytochrome P450 epoxygenases P450 (CYP) [17]. COX-1 and-2 convert AA to prostaglandin H_2_ (PGH_2_), the precursor of the series-2 prostanoids, which are produced by a series of specific synthases during cell-specific conversion processes. The LOX enzymes 5-, 12- and 15-LOX convert AA to hydroperoxy eicosatetraenic acids (HpETEs), which are subsequently reduced to 5(S)-, 12(S)- and 15(S)-HETE (hydroxyeicosatetraenoic acid), respectively. Unlike other lipoxygenases, 5-LOX requires the presence of the 5-lipoxygenase activating protein (FLAP) for productive leukotriene synthesis *in vivo* [11]. The HETEs are further reduced by hydroxyeicosatetraenoic dehydrogenase (HEDH) to oxo-eicosatetraenoic acids (oxo-ETE). Cytochrome P450 converts AA to epoxyeicosatrienoic acid (EET) regioisomers, which in turn are converted by soluble epoxide hydrolase (sEH) to dihydroxyeicosatrienoic acids (DiHETrEs) [18].

Linoleic acid (AA) is an essential PUFA and its metabolism follows pathways regulated by LOX and CYP enzymes. The LOX pathway (involving 5- and 15-LOX) is responsible for oxidation of LA to hydroperoxyoctadecadienoic acids (HpODE), which are then converted to hydroxyoctadecadienoic acids (HODEs) and further to oxo-octadecadienoic acids (oxo-ODE). 9(S)-HODE, 9(S)-HpODE and 13(S)-HpODE can also be converted to trihydroxyoctadecenoic acids (9,12,13-TriHOME, 9,10,13-TriHOME). The CYP pathway leads to epoxyoctadecamonoenoic acids (EpOME), which are subsequently converted to dihydroxyoctadeca(mono)enoic acids (DiHOME).

## Materials and Methods

### Study design

Details of the study have been previously described [5]. In brief, 12 healthy adults (six males, six females; age, 54 ± 6 years; BMI, 22 ± 1 kg/m^2^; and waist circumference, 80 ± 8 cm) participated in a crossover study including four test days separated by six washout days each. On each test day, a different dietary challenge was tested. One of the dietary challenges was an oral glucose tolerance test (OGTT), in which the subjects consumed 75 g of glucose (300 kcal) dissolved in 300 ml of water.

On each test day, the subjects arrived at the research facility in the morning after an overnight fast of 10 hours. After the collection of baseline blood samples (t = 0), subjects consumed one of the challenge test products within 10 min. Blood samples for oxylipin analysis were collected at regular time points (0, 2, 4, 6, 8, 10 h) up to 10 h. No other foods or beverages were allowed during the day, except water.

The study protocol was approved by the independent Medical Ethics Committee of TNO METOPP (Tilburg, the Netherlands) and all subjects gave written informed consent. Data is available through the Nutritional Phenotype database (http://studies.dbnp.org/ADMIT_01) [19].

### Experimental methods

#### Sample collection and preparation

Blood samples were collected in tubes containing EDTA as anticoagulant. Whole blood was centrifuged for 10 min at 4°C at 13,000 rpm within 15 min of collection. Serum and plasma were immediately separated, aliquoted and stored at -80°C. For the oxylipin analysis, an inhibitor cocktail consisting of butylated hydroxytoluene, indomethacin, paraoxon and phenylmethylsulfonyl fluoride was directly added to the plasma samples (250 μL) before storage.

#### LC-MS/MS analysis

Oxylipins were analyzed on a UPLC coupled to a Xevo TQ-S mass spectrometer (Waters) according to a previously described procedure [9,20]. In short, methanol extracts of 250 μl of plasma sample were concentrated using solid-phase extraction columns. The concentrated elutes were injected on a UPLC column and separated using gradient elution. The gradient started with 95% A (MQ water with 0.1% FA) and 5% B (ACN with 0.1% FA) followed by a linear increase to 70% A and 30% B. This was followed by a linear increase towards 50% B, and subsequently a switch to 100% B, which was maintained until the end of the run. The column was maintained at 323.15 K during analysis, and the samples were kept at 283.15. The MS was operating in selective reaction mode using electrospray ionization in negative ion mode. Peak identification and quantification were performed using the MassLynx software version 4.1. All calibration curves were run in duplicate, from which one regression equation was generated. Quality control samples were included in each analytical run to check the quality of the analysis and to correct for accuracy. From the comprehensive profile of oxylipins that was covered by the LC-MS/MS platform only 22 oxylipins were detected in an initial batch of pooled samples. Hence only these oxylipins were selected for further processing.

### Modeling techniques

#### Network simplification

We focused on AA and LA metabolism, a subset of the larger oxylipin metabolic network. The metabolic routes of AA and LA were lumped to a simplified network to include only the metabolites that showed a clear response upon OGTT according to the following three rules, as commonly done in chemical reaction kinetics, metabolic engineering and systems biology [21,22]:

1. Multistep reactions with unmeasured intermediate metabolites were simplified to a single-step reaction, for instance, the multistep reaction AA → 5-HpETE → 5-HETE is lumped to AA → 5(S)-HETE;
2. Multistep reactions with intermediate metabolites that did not show any variation were simplified to a single-step reaction, for instance, the reaction AA → 11,12-EET → 12,12-DiHETrE is lumped to AA → 12,12-DiHETrE as 11,12-EET showed no or little variation;
3. Metabolites involved in unmeasured branches of the metabolic pathways were lumped to a generic degradation reaction, for instance, compounds originating from 5(S)-HETE are set to unmeasured in our lumped network.

**Fig 1:**
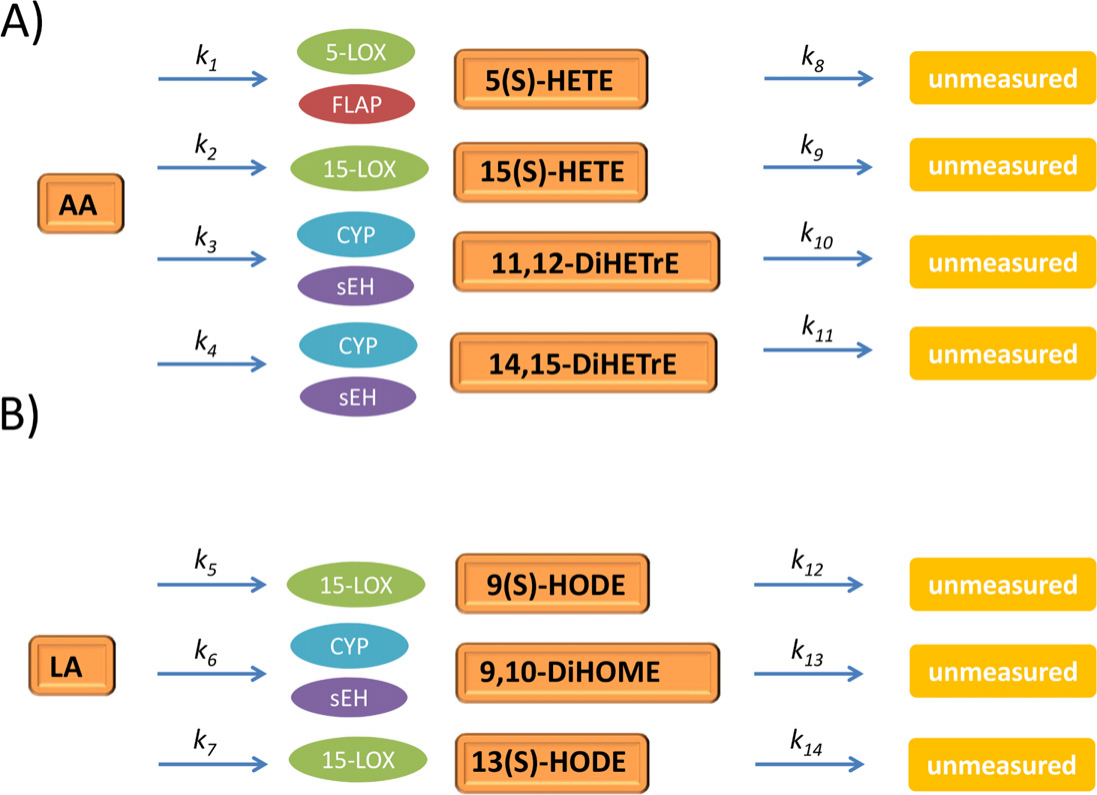
Lumped networks for oxylipins metabolism. Lumped networks for arachidonic (panel A) and linoleic (panel B) acid metabolism. Abbreviations: Dihydroxyeicosatrienic acid (DiHETrE); Dihydroxyoctadecenoic acid (DiHOME); Hydroxyeicosatetraenoic acid (HETE); Hydroxyoctadienoic acid (HODE). The rationale underlying the lumping strategy is described in Section: Network simplification.

The resulting lumped networks for AA and LA are shown in Fig. 1, panels A and B respectively. Enzymes regulating metabolite degradation were not measured.

#### Data overview

**Fig 2:**
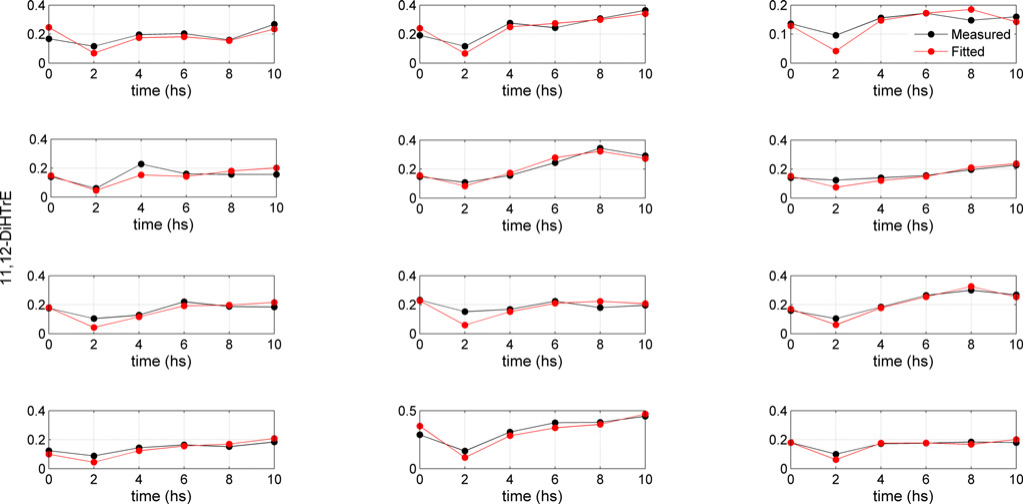
Concentration profiles for 11,12-DiHeTrE. Measured (black dots) and fitted (red dots) time concentration profiles for 11,12-DiHeTrE (AA metabolism) for 12 subjects. The regression parameter *A*, lumping the coefficients *P* and *D* (dimension t^-1^) describing the production rate and the degradation rate for 11,12-DiHeTrE (see Equation (3)) is obtained under a pseudo-steady-state approximation (Equation (4)) and obtained via Equation (7).

**Fig 3:**
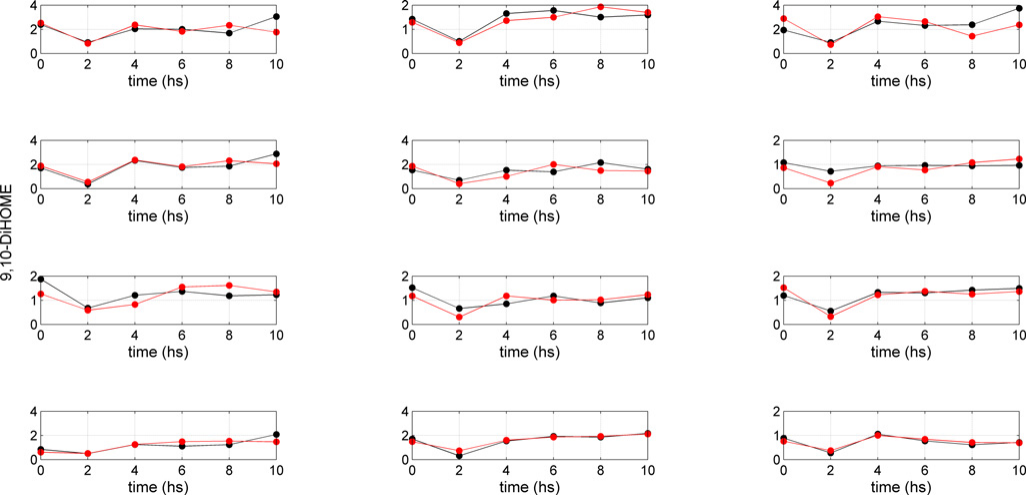
Concentration time profiles for 9,10-DiHOME. Measured (black dots) and fitted (red dots) time concentration profiles for 9,10-DiHOME (AA metabolism) for 12 subjects. The regression parameter *A*, lumping the coefficients *P* and *D* (dimension t^-1^) describing the production rate and the degradation rate for 9,10-DiHOME (see Equation (3)) is obtained under a pseudo-steady-state approximation (Equation (4)) and obtained via Equation (7).

The lumping of the metabolic networks of AA and LA leads to a simplified version of the metabolic networks where four metabolites (5(S)-HETE; 15(S)-HETE; 11,12-DiHETrE; 14,15-DiHTrE) were retained for AA, and three for LA (9(S)-HODE; 9,10-DiHOME; 13(S)-HODE). The study design involved 12 subjects from whom blood samples for oxylipin analysis were taken at regular time points (0, 2, 4, 6, 8, 10 h). Fig. 2 and 3 provide an overview of the changes in concentration of 11,12-DiHETrE and 9,10-DiHOME over time for the 12 subjects, respectively. Although the time curves follow similar patterns, differences between the subjects can be observed. Data covers a relatively long time period, but it should be noted that information is not evenly spread over the eight time points. Only the second measurement at 2 h strongly differed from the rest of the measurements. Modeling this kind of data characterized by a paucity of information is challenging; the following sections propose a strategy to tackle this problem.

### Mathematical models

A deterministic model in the form of a mathematical function-based model is devised whose mathematical parameters are to be estimated from the experimental data available. The mathematical model must be conceived on the basis of a priori biological knowledge in a way that the parameters can be estimated from the experimental data available. In the case of AA and LA metabolism, the biochemical process is known and described by the network structure of a degradation pathway that can be translated into a system of mathematical (kinetic) equations. Depending on the assumptions, different mathematical models can be considered.

#### Model 1: System of ordinary differential equations

Given the underlying biochemistry, the model is given by a system of ordinary differential equations (ODEs). With reference to panels A and B of Fig. 1, the corresponding systems of ODEs for the AA metabolic pathway are given by the equations

$$\left\{ \begin{array}{l} \frac{d[\text{5(S)-HETE}]}{dt}=k_{1}[\text{AA}]-k_{8}[\text{5(S)-HETE}]\\ \frac{d[\text{15(S)-HETE}]}{dt}=k_{2}[\text{AA}]-k_{9}[\text{15(S)-HETE}]\\ \frac{d[\text{11,12-DiHETrE}]}{dt}=k_{3}[\text{AA}]-k_{10}[\text{11,12-DiHETrE}]\\ \frac{d[14,\text{15-DiHETrE}]}{dt}=k_{4}[\text{AA}]-k_{11}[14,\text{15-DiHETrE}] \end{array}\right.$$ (1)

and for LA by

$$\left\{ \begin{array}{l} \frac{d[\text{9(S)-HODE}]}{dt}=k_{5}[L\text{A}]-k_{12}[\text{9(S)-HODE}]\\ \frac{d[\text{9,10-DiHOME}]}{dt}=k_{6}[L\text{A}]-k_{13}[\text{9,10-DiHOME}]\\ \frac{d[\text{13(S)-HODE}]}{dt}=k_{7}[\text{LA}]-k_{14}[\text{13(S)-HODE}] \end{array}\right.$$ (2)

Here [*x*] indicates the concentration of metabolite *x*. Parameters k_1_ to k_7_ describe the conversion of AA and LA to the corresponding downstream metabolites. Parameters k_8_ to k_14_ describe the degradation of these compounds into further downstream metabolites that were not measured. Reaction parameters are summarized in Table 1. Altogether Model 1 describes the AA and LA metabolic branches as first-order kinetics. As each parameter appears only in one equation, the parameters can be estimated by solving independently each one of the seven ODEs. In other words, Equation (1) represents an uncoupled system of ODEs.

**Table 1:** Summary of the reactions and parameters involved in the lumped AA and LA metabolism under investigation.

No.	Reaction	Control enzyme	Parameter name
**1**	AA → 5(S)-HETE	5-LOX, FLAP	*k*_*1*_
**2**	AA → 15(S)-HETE	15-LOX	*k*_*2*_
**3**	AA → 11,12-DiHETrE	CYP, sEH	*k*_*3*_
**4**	AA → 14,15-DiHETrE	CYP, sEH	*k*_*4*_
**5**	LA → 9(S)-HODE	15-LOX	*k*_*5*_
**6**	LA → 9,10-DiHOME	CYP, sEH	*k*_*6*_
**7**	LA → 13(S)-HODE	15-LOX	*k*_*7*_
**8**	5(S)-HETE→		*k*_*8*_
**9**	15(S)-HETE →		*k*_*9*_
**10**	11,12-DiHETrE →		*k*_*10*_
**11**	14,15-DiHETrE →		*k*_*11*_
**12**	9(S)-HODE →		*k*_*12*_
**13**	9,10-DiHOME →		*k*_*13*_
**14**	13(S)-HODE →		*k*_*14*_

#### Model 2: Pseudo-steady-state model

Model 1 can be simplified assuming a pseudo steady state [23]: The two processes of AA and LA conversion in the white blood cells and the dynamics of the blood AA and LA concentration happen on different time scales—hours for the blood dynamics and seconds for the degradation of AA and LA in the cells. For this reason, the variation of the downstream products can be considered to be negligible, at each given measurement point [24]. This is equivalent to setting the time derivative term *d/dt* in Equations (1) and (2) to 0.

The pseudo-steady-state approximation allows the use of algebraic equations instead of differential equations to estimate the reaction parameters starting from the measured concentration of the chemical species considered. For the sake of simplicity we will indicate with *n(t)* the concentration at time *t* of either AA or LA and with *y(t)* the concentration of any of their downstream metabolites (see Equations (1) and (2)) at time *t*, so that we can write

$$\frac{dy}{dt}=P\cdot n(t)-D\cdot y(t)$$ (3)

where the coefficients *P* and *D* (dimension t^-1^) are the production rate and the degradation rate for compound *y*, respectively. In the pseudo-steady-state approximation Equation (3) becomes

0=P⋅n(t)−D⋅y(t) (4)

From Equation (4) it follows that

$$y(t)=\frac{P}{D}n(t)$$ (5)

that is

y(t)=A⋅n(t) (6)

where we have set *A = P/D*, thus the ratio of the production and degradation rates of compound y.

The problem is now reduced to the estimation of a single parameter *A*. This approach enables the complexity of the model to be reduced by reducing the number of parameters to be estimated from two to one as *P* and *D* are not estimated separately but only their ratio: For instance, in the reaction involving the conversion of AA to 5(S)-HETE and its subsequent degradations (see Fig. 1, panel A and Table 1), the two parameters controlling the reaction, *k*_*1*_ and *k*_*8*_, are not estimated separately but their k_1_/k_8_ ratio is estimated.

It should be noted that the pseudo-steady-state approximation leading to Equation (6) implies that y *(t)* and *n (t)* are correlated. This means that this approximation is justified if, and only if, the concentrations of AA and LA are correlated with those of their downstream metabolites. As shown in Tables 2 and 3, a strong correlation generally exists between the concentrations of AA and LA and their metabolites for all 12 subjects in the study.

**Table 2:** Correlation values of arachidonic acid (AA) and its downstream metabolites based on six time points for the 12 subjects in the study.

Subject	5(S)-HETE *vs* AA	15(S)-HETE *vs* AA	11,12-DiHETrE *vs* AA	14,15-DiHETrE *vs* AA
**1**	0.98	0.83	0.72	0.79
**2**	0.75	0.92	0.92	0.93
**3**	0.57	0.59	0.89	0.83
**4**	0.86	0.80	0.70	0.69
**5**	0.71	0.76	0.96	0.92
**6**	0.25	0.69	0.95	0.93
**7**	0.67	0.90	0.91	0.86
**8**	0.84	0.82	0.78	0.69
**9**	0.88	0.91	0.98	0.91
**10**	0.66	0.70	0.98	0.86
**11**	0.98	0.86	0.93	0.93
**12**	0.84	0.65	0.97	0.89

The pseudo-steady-state approximation implies that AA and its four downstream metabolites are correlated as given by Equation (6) in the text.

**Table 3:** Correlation values of linoleic acid (LA) and its downstream metabolites based on six time points for the 12 subjects in the study.

Subject	9(S)-HODE *vs* LA	9,10-DiHOME *vs* LA	13(S)-HODE *vs* LA
**1**	0.61	0.51	0.65
**2**	0.92	0.85	0.91
**3**	0.60	0.55	0.61
**4**	0.81	0.86	0.83
**5**	0.64	0.58	0.65
**6**	0.93	0.73	0.87
**7**	0.78	0.53	0.72
**8**	0.75	0.63	0.81
**9**	0.85	0.89	0.89
**10**	0.69	0.74	0.74
**11**	0.99	0.97	0.98
**12**	0.88	0.95	0.90

The pseudo-steady-state approximation implies that LA and its four downstream metabolites are correlated as given by Equation (6) in the text.

#### Parameter estimation for Model 1

In general, to be of any practical use, the model parameters must be determined with sufficient precision to adequately describe the inter-individual differences in the relative activity of their AA and LA metabolism. The system of differential equations can be solved numerically using standard procedures such as the standard ODE45 solver (MATLAB 2012b, The MathWorks, Inc., Natick, MA, US). As mentioned earlier, the systems are uncoupled permitting simple estimation procedures. During this fitting process, very large correlations were observed between the estimates of the two parameters pertaining to a single equation; these estimates were therefore deemed unreliable and not considered for further analysis. Instead, Model 2 was used.

#### Parameter estimation for the pseudo-steady-state model

Model 2 requires only the estimation of one parameter: *A*. This can be easily done by regressing *y(t)* on *n(t)*. The least-squares solution for A, that is its estimate *Â*, is given by

$$\hat{A}={\left(n^{T}n\right)}^{-1}(n^{T}y)$$ (7)

A measure of the precision of the linear regression estimate *Ĉ* can be expressed in terms of confidence intervals (CIs). The CIs for the linear regression are given by

$$\hat{A}\pm t(1-\frac{\alpha }{2},n-p)SE(\hat{A})$$ (8)

where *SE(Â)* is the standard error of the coefficient estimate, and *t(1–α/2*,*n—p)* is the 100(1–α/2) percentile of *t*-distribution with *n—p* degrees of freedom; *n* is the number of observations and *p* is the number of regression coefficients to be estimated. In the present case *n = 6* and *p = 1*.

#### Interpretation of the A parameter

The *A* parameter is defined as the ratio of the production rate *P* and the degradation rate *D* for compound *y* (see Equations 5 and 6). For each branch, *A* can be considered as a summary of the information about a) the enzymatic process controlling the conversion of AA or LA to a specific metabolite, and b) the (possibly enzymatic) further downstream process that leads to the degradation of this specific metabolite to unmeasured compounds. Altogether *A* can be viewed as a measure of the *relative* activity of a given branch of the metabolic pathway of either AA or LA. Nothing can be said about the absolute values of *P* and *D* parameters because the relative nature of the *A* parameter is not able to describe scale factors between *P* and *D* (see Equations (4) and (5)). Nonetheless, differences in the values of the *A* parameter describe inter-individual differences in the relative activity of these metabolic branches.

#### Comparison of A parameters

Once *A* parameters are obtained for all subjects and for all branches of the AA and LA pathways, two kinds of comparison are of interest:

1. Comparing the A parameters of the different branches of the AA and LA pathways within a subject, and
2. Given a branch of AA or LA pathway, comparing the corresponding *A* parameters across subjects.

These two comparisons are intended to provide information about 1) the differential relative activity of different branches of the AA/LA pathways within a subject, and 2) inter-individual variability of the relative activity of a specific branch across different subjects. To compare the relative activity of the different branches (comparison 1), the ratios of the corresponding *A* parameters can be considered, leading to a grand total of six ratios ( = 4(4–1)/2) for the AA pathway and three ratios for the LA pathway.

In the jargon of pharmacokinetics and pharmacodynamics, reaction phenotyping measures the proportion of metabolism that is carried out by different enzymes [25]. In light of this, the use of the *A* parameters seems an appropriate choice to quantify the relative activity of different branches of a metabolic pathway.

The phenotypic parameter *A*, for the AA and LA metabolic pathways for 12 subjects, obtained through Model 2, is given in Table 4 (for AA) and Table 5 (for LA) with the associated 95% confidence intervals. Fitted curves (using Equation 13) for two selected compounds (11,12-DiHTrE and 9,10-DiHOME) are shown in Figs. 2 and 3. Fitted curves for the other compounds are given in file S1 File.

**Table 4:** Estimates of the *A* parameter for Model 2 obtained by linear regression for the four branches of the AA metabolism: 5(S)-HETE, 15(S)-HETE, 11,12-DiHETrE and 14,15-DiHETrE.

Subject	5(S)-HETE	15(S)-HETE	11,12-DiHETrE	14,15-DiHETrE
**1**	0.62 ± 0.08	1.08 ± 0.16	1.62 ± 0.41	1.11 ± 0.25
**2**	0.58 ± 0.22	1.03 ± 0.06	1.91 ± 0.29	1.37 ± 0.24
**3**	1.01 ± 0.32	0.99 ± 0.23	1.77 ± 0.33	1.26 ± 0.20
**4**	0.91 ± 0.16	1.31 ± 0.26	1.51 ± 0.43	1.03 ± 0.27
**5**	0.60 ± 0.14	1.24 ± 0.38	2.09 ± 0.21	1.27 ± 0.20
**6**	0.56 ± 0.22	0.81 ± 0.23	1.88 ± 0.30	1.24 ± 0.28
**7**	0.99 ± 0.31	0.91 ± 0.17	1.50 ± 0.32	0.92 ± 0.22
**8**	1.12 ± 0.24	1.05 ± 0.16	1.95 ± 0.48	1.05 ± 0.26
**9**	0.73 ± 0.13	0.80 ± 0.13	1.76 ± 0.16	1.24 ± 0.20
**10**	0.74 ± 0.22	0.78 ± 0.17	1.62 ± 0.33	1.02 ± 0.30
**11**	0.90 ± 0.05	0.75 ± 0.14	2.21 ± 0.33	1.23 ± 0.19
**12**	1.06 ± 0.23	1.08 ± 0.23	1.55 ± 0.18	0.93 ± 0.16

Estimates are given together with the associated 95% confidence interval indicated by the lower (Lb) and upper (Ub) bound calculated as given by Equation (14) in the text. *A* parameters are × 10^4^.

**Table 5:** Estimates of the *A* parameter for Model 2 obtained by linear regression for the three branches of the LA metabolism: 9(S)-HODE, 9–10-DiHOME and 13(S)-HODE.

Subject	9(S)-HODE	9,10-DiHOME	13(S)-HODE
**1**	0.59 ± 0.15	0.31 ± 0.07	0.59 ± 0.13
**2**	0.97 ± 0.13	0.34 ± 0.05	1.10 ± 0.14
**3**	0.53 ± 0.22	0.38 ± 0.15	0.53 ± 0.19
**4**	0.87 ± 0.19	0.42 ± 0.07	0.96 ± 0.21
**5**	0.37 ± 0.15	0.26 ± 0.07	0.41 ± 0.16
**6**	0.25 ± 0.04	0.15 ± 0.05	0.28 ± 0.05
**7**	0.62 ± 0.12	0.26 ± 0.08	0.61 ± 0.15
**8**	0.67 ± 0.18	0.24 ± 0.07	0.66 ± 0.16
**9**	0.44 ± 0.09	0.26 ± 0.04	0.48 ± 0.09
**10**	0.63 ± 0.20	0.31 ± 0.09	0.70 ± 0.22
**11**	0.92 ± 0.15	0.33 ± 0.04	0.98 ± 0.16
**12**	0.57 ± 0.14	0.17 ± 0.03	0.55 ± 0.13

Estimates are given together with the associated 95% confidence interval indicated by the lower (Lb) and upper (Ub) bound calculated as given by Equation (14) in the text. *A* parameters are ×10.

It is important to note that the concentrations of AA are roughly one order of magnitude larger than those of LA, while the concentrations of the AA metabolites are roughly one order of magnitude smaller than those of the LA metabolites. Thus the *A* parameters of the AA metabolites are 10^2^ to 10^3^ times higher than those of the LA metabolites.

In general, if within a subject an A parameter is larger for one metabolite (e.g. 11,12-DiHETrE) than for another metabolite (e.g. 5(S)-HETE), this means that either the reaction rate constant *k*_*3*_ for the production (see Table 1) is larger or the reaction rate constant *k*_*10*_ for the degradation of 11,12-DiHTrE is smaller than the respective reaction rate constants for 5(S)-HETE. Since both 11,12-DiHTrE and 5(S)-HETE are produced from AA, 11,12-DiHETrE is more abundant than 5(S)-HETE.

For comparison across the subjects, metabolite concentrations cannot be directly derived from the A parameters due to different substrate (LA or AA) concentrations. Yet the pathway activities can still be compared between the subjects by setting the *A* parameters in proportion to each other. For example, for subject 11 the *A* parameters and thus the concentrations of 15-HETE and 11,12-DiHTrE differ by a factor of 3, while for subject 4 the *A* parameters of 15-HETE and 11,12-DiHTrE are similar, meaning that 15(S)-HETE is three times less abundant in subject 11 and present in approximately equal amounts in subject 4, relative to the production of 11,12-DiHETrE.

#### Multivariate analysis

To highlight patterns of (dis)similarity among the subjects, principal component analysis (PCA) was performed on the **R** matrix obtained by concatenating the matrices of the *A* parameter estimations for the 12 subjects for both AA and LA metabolic pathways (i.e. data in Tables 4 and 5). The **R** matrix has the size 12 × (4 + 3), subjects × variables. As the *A* parameters are obtained from the ratio of two quantities (see Equation 6), the logarithm of **R** was taken for linearization purposes [26]. The columns of the transformed matrix were centered to zero mean and scaled to unit variance before PCA. A scree plot [27] test was used to determine the optimal number of components (two in this case, 70% of variance explained).

## Results and Discussion

Human plasma samples were collected during an OGTT in a previous study and the responses of LA, AA and their oxidation products were detected in a quantitative manner [9]. The OGTT induced changes in several oxylipins that are involved in LOX and CYP pathways, namely in 5(S)-HETE, 15(S)-HETE,11,12-DiHETrE and 14,15-DiHETrE, which are derived from AA, and in 9(S)-HODE, 13(S)-HODE and 9,10-DiHOME, which are derived from LA. These metabolites may play a role in endothelial inflammation and vascular function. In contrast, OGTT did not induce changes in oxylipins that are involved in the COX pathway.

Following OGTT, significant reductions in AA, LA and their downstream metabolites were observed after 2 h when compared to baseline and the water control challenge. The concentrations reached control levels after 4–6 h (Figs. 2 and 3 and supplemental file S1 File). The initial reductions in oxylipins were explained by the reduced release of fatty acids from adipose tissue and triacylglycerides as a result of the postprandial increase in insulin. As soon as insulin declines, fatty acids are again released into plasma, most likely leading to increases in oxylipins after 2 hours [13,28]. Significant inter-individual differences in the response of the different oxylipins upon the OGTT [9] were observed, and we selected this study to derive quantitative descriptors of phenotypical pathway preferences.

To arrive at phenotypical descriptors of oxylipin metabolism we built a regression model that incorporates both the time-resolved oxylipin concentrations and the a priori knowledge on the degradation pathways of AA and LA. This was accomplished by first lumping the metabolic networks of interest. Lumping is a common approach to reducing the dimensionality and the complexity of a model by preserving its physiological nature in such a way that no useful information about underlying biological processes is lost [29]. The lumped metabolic networks are given in Fig. 1.

We obtained phenotypic parameters by regressing, for each individual, the AA and LA concentration profiles on the corresponding byproducts concentration levels, assuming a pseudo-steady-state: in this way the problem reduced to the estimation of a single parameter *A*. The combination of two or more parameters is a well-known strategy to overcome the problem of unidentifiable parameters [8,30], and we used it to characterize individual phenotypical variation of the oxylipins metabolism.

We found that the *A* parameters between the AA and LA metabolites differed by 2 to 3 orders of magnitude due to higher concentrations of AA when compared to LA and lower concentrations of the AA metabolites when compared to the LA metabolites. Thus, LA metabolites are in general more efficiently produced than AA metabolites.

**Fig 4:**
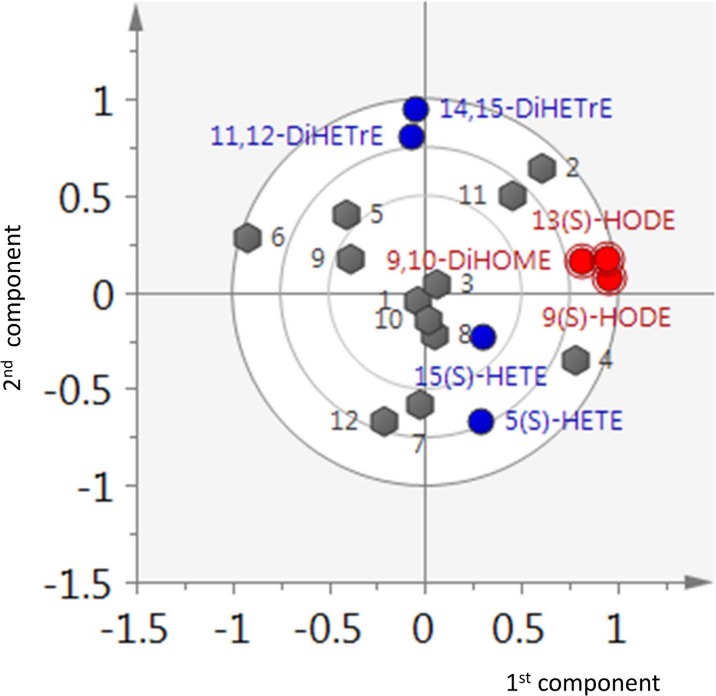
Principal component analysis of the AA and LA metabolic branches. Biplot for the principal component analysis of AA and LA branches of the oxylipins metabolism. The first and second components respectively describe the LA and AA pathways. It should be noted that the 12 subjects tend to spread either along the first component (LA metabolism) or along the second component (AA metabolism).

**Fig 5:**
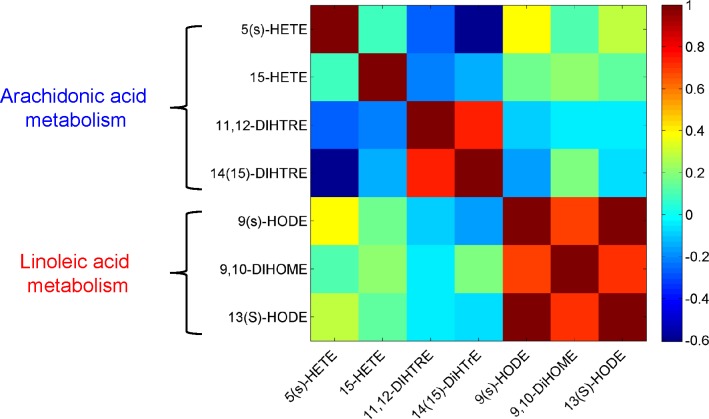
Correlation among AA and LA parameters. Heat-map for the correlation matrix among the parameters describing AA and LA downstream metabolism, obtained using the estimated values obtained for the seven *A* parameters for the 12 subjects. There is no (or very weak) correlations between the AA and LA metabolites, suggesting that the formation of AA and LA metabolites is not related to each other.

To get an overview of the pathway-related and phenotypic differences in the AA and LA metabolism, principal component analysis (PCA) was performed on the phenotypic parameters *A* that were calculated for the AA (Table 4) and LA (Table 5) metabolites for each of the 12 subjects. From the biplot in Fig. 4 it becomes apparent that the first component is exclusively described by the LA metabolites, while the second component mostly explains the variation in AA metabolites. This indicates that the AA and LA pathways are independent. Also the correlation matrix (Fig. 5) calculated on the basis of the *A* parameters of the AA and LA metabolites across the subjects shows no strong correlations between the AA and LA metabolites, thus confirming that the formation of AA and LA metabolites is not related to each other. This is surprising considering that the same enzymes (LOX, CYP) are involved in the two pathways. For example, 15(S)-HETE and 13(S)-HODE are formed through the activity of the 15-LOX enzyme from AA and LA, respectively. Similarly, the CYP enzyme regulates the formation of 9,10-DiHOME from LA and 11,12-DiHETrE and 14,15-DiHETrE from AA.

We observed a strong inter-individual variation in metabolizing LA with respect to AA. In contrast, we observed strong correlations between the LA metabolites, meaning that all subjects metabolized LA in a similar manner, irrespective of the enzyme. (The stronger correlation between the LOX metabolites than between the LOX and CYP metabolites may still be important to note.) In comparison to the LA metabolism, we only found a strong positive correlation between the CYP metabolites of AA and no correlation between the LOX metabolites of AA. This indicates that AA is metabolized using a common route through the activity of the CYP enzyme, while the use of the LOX enzymes is subject to inter-individual variation. The negative correlations between the CYP and LOX metabolites of AA suggest competition between the CYP and LOX pathways.

When ranking the *A* parameters of the metabolites for each subject within the AA and LA pathways, respectively, differences between the relative preferences of the AA and LA metabolism were noted. Regarding the AA metabolism, 11,12-DiHETrE had the largest A parameter for each subject, followed by 14,15-DiHETrE and 15(S)-HETE. 5(S)-HETE in general had the lowest *A* parameter, except for subjects 7 and 8. These results show (i) that there is an overall preference for producing 11,12-DiHETrE and a particular preference for producing 11,12-DiHETrE over 14,15-DiHETrE within the CYP pathway, and (ii) that the formation of 5(S)-HETE from AA through the 5-LOX activity is the least preferred pathway for AA metabolism. This order of relative pathway activity is opposed to the order observed for LA. For each subject, the *A* parameters of 9(S)-HODE and 13(S)-HODE were larger than those of 9,10-DiHOME, showing that the LOX pathways are preferred over the CYP pathway.

Moreover, we noticed significant inter-individual variation in the LA and AA metabolism. While the individual time curves of the AA and LA and their metabolites (Figs. 2 and 3) appear similar, the *A* parameters clearly reveal differences between the subjects. It appears that subjects 1, 3, 8 and 10 metabolized LA and AA in a similar manner, while stronger differences in the metabolism patterns were observed for the other subjects. For example, subject 2 produced more 14,15-DiHETrE, 9(S)-HODE and 13(S)-HODE, while subject 12 produced more 5(S)-HETE relative to all subjects and metabolites. These inter-individual variations in the *A* parameters account for differences in both pathway activity and substrate (LA and AA) concentrations. Inter-individual differences were also observed for the AA and LA metabolism, respectively. With regard to the LA metabolism, the *A* parameters for 9(S)-HODE and 13(S)-HODE are 1.4–3.4 times higher when compared to 9,10-DiHOME. The largest difference was observed for subjects 12 and 3. While subject 12 produced three times more 9(S)-HODE and 13(S)-HODE than 9,10-DiHOME, subject 3 produced approximately equal amounts of these metabolites. With regard to the AA metabolism, 5(S)-HETE, 15(S)-HETE and 14,15-DiHETrE were 1.5–3.5, 1.5–3 and 1.4–1.9 times less abundant than 11,12-DiHETre, thus showing the strongest inter-individual variation for the two LOX metabolites (S)-HETE and 15(S)-HETE. For example, subjects 5 and 6 produced 3.5 times less 5(S)-HETE, while subjects 12 and 7 produced only slightly less of this metabolite when compared to 11,12-DiHETrE. In comparison, subjects 4 and 11 displayed the smallest and largest difference for 15(S)-HETE relative to 11,12-DiHETrE.

## Conclusions

By incorporating a priori knowledge on oxylipin pathways we were able to derive a phenotypical descriptor that captures the inter-individual differences in AA and LA metabolism in response to an OGTT. This was accomplished by defining the *A* parameter that summarizes information on the enzymatic processes controlling AA and LA degradation. The simplification of the metabolic network and the assumption of a pseudo-steady-state model enable the modeling of a sparsely sampled response with a straightforward linear regression approach. Multivariate analysis and correlation analysis shows that there is little or no competition between the LA and AA pathways, although the same enzymes are shared between the two pathways. Yet, the *A* parameters suggest that within the LA and AA pathways AA is preferably metabolized to the CYP metabolites (in particular 11,12-DiHETrE), while the LOX pathways are preferred over the CYP pathway for LA. Comparison of the phenotypic *A* parameters suggests that the subjects can be stratified on the basis of preferred relative activity of the AA and LA metabolism.

The method used to derive all the conclusions presented here is linear regression, which is well understood and easy to apply. But it can only be applied if the pseudo-steady-state assumption holds. If this is not the case then much more complicated numerical approaches must be used. These approaches are also more demanding in terms of the data quality than linear regression, but can in principle be used in a similar strategy. In cases where the pseudo-steady-state assumption was valid, linear regression provided biological insight into the variability of oxylipin responses.

## Supporting Information

S1 File Supporting Information Figure. Plots of measured of and fitted concentration for AA and LA byproducts.
(PDF)
