# Supplementary figures and images for: Strategies for Individual Phenotyping of Linoleic and Arachidonic Acid Metabolism Using an Oral Glucose Tolerance Test
Source: PLoS One. 2015 Mar 18;10(3):e0119856. doi: 10.1371/journal.pone.0119856 (PMC4364740; doi:10.1371/journal.pone.0119856)

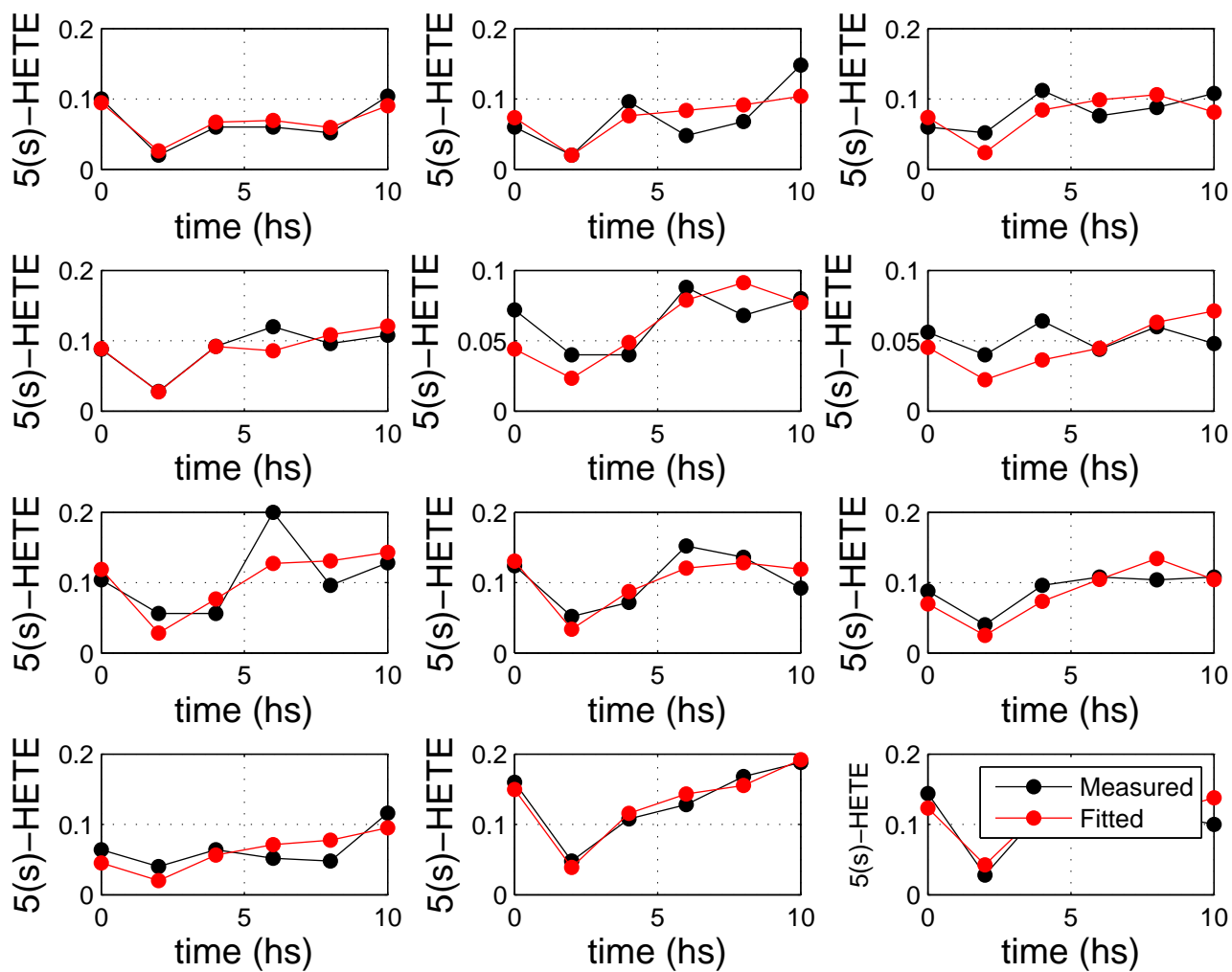

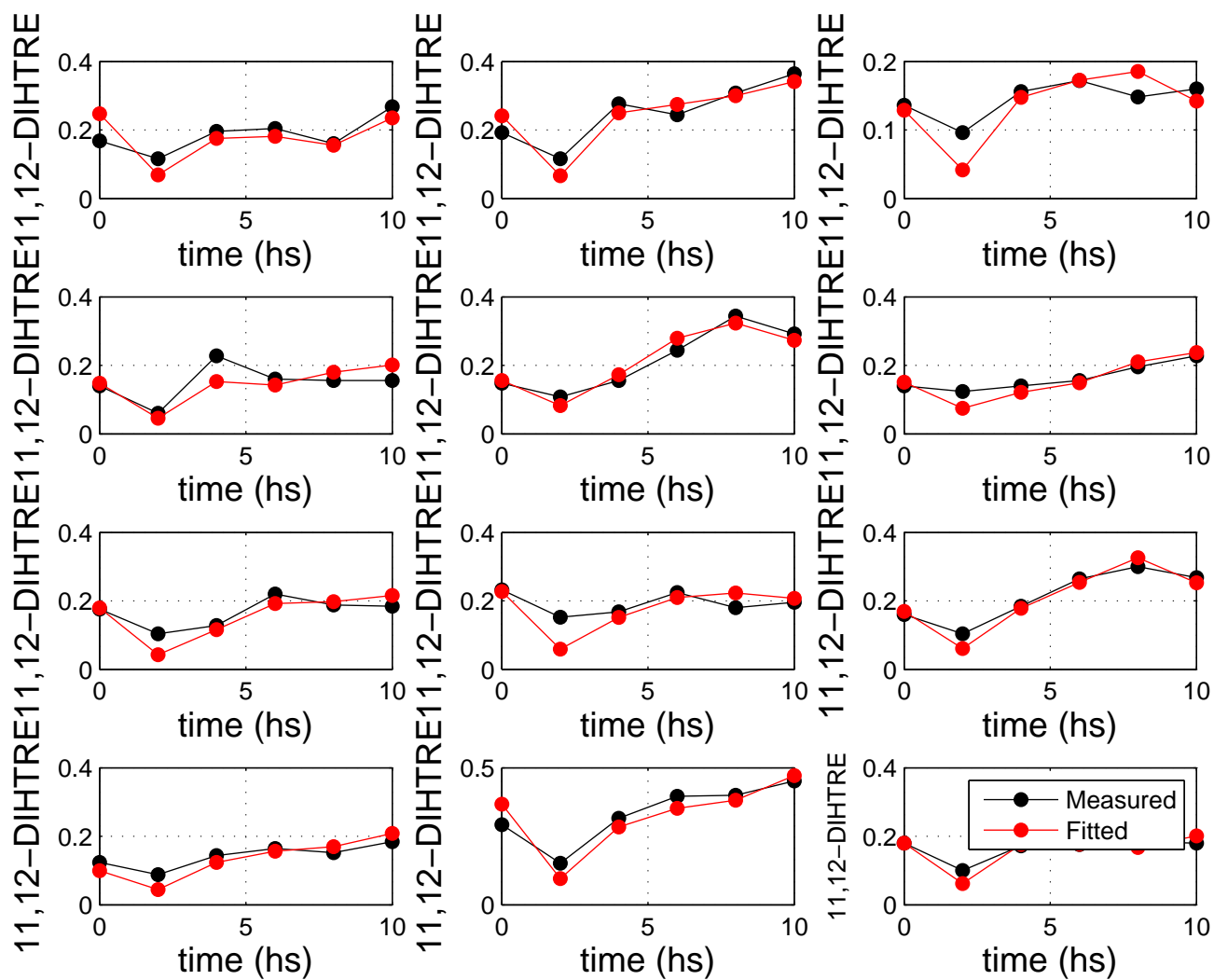

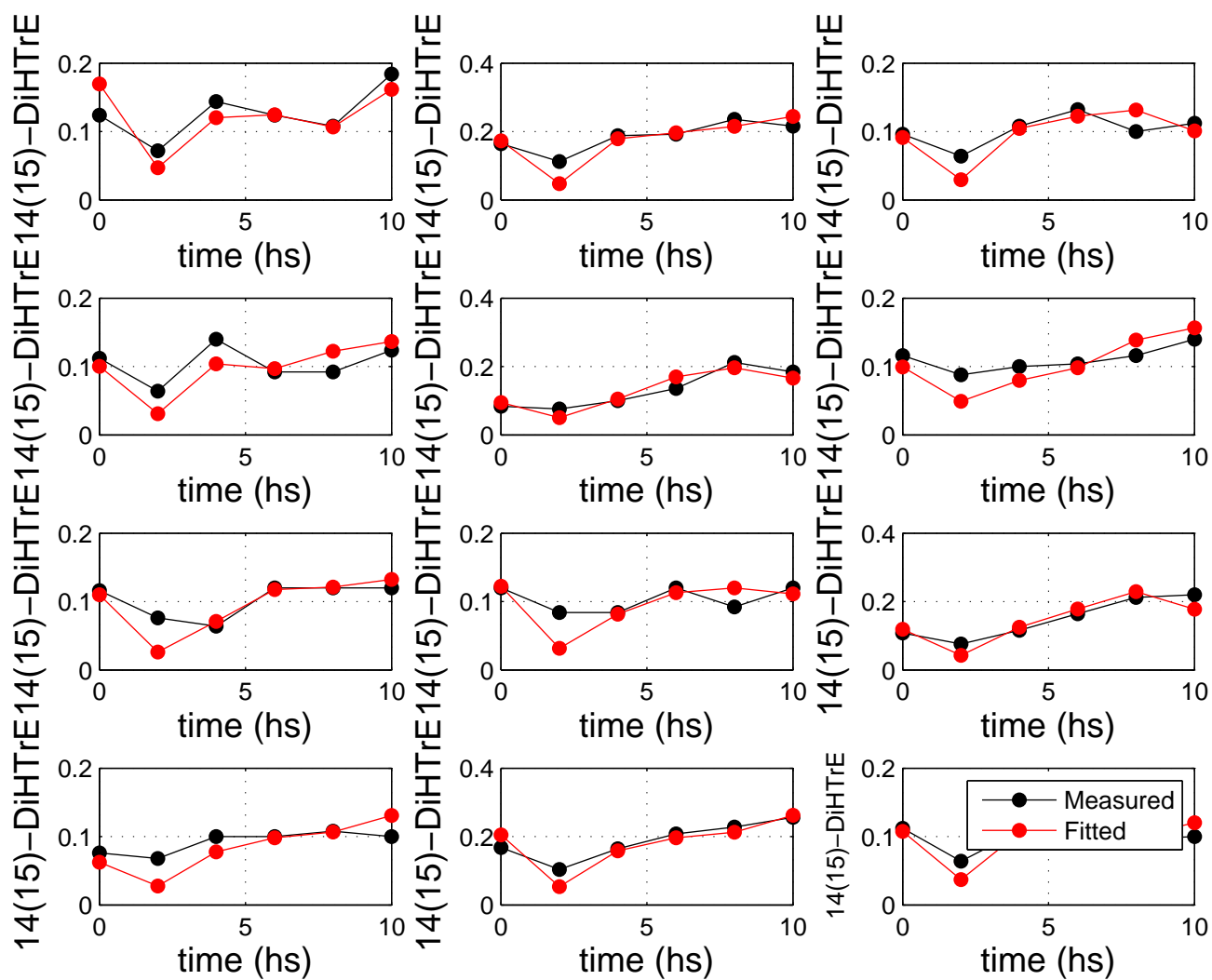

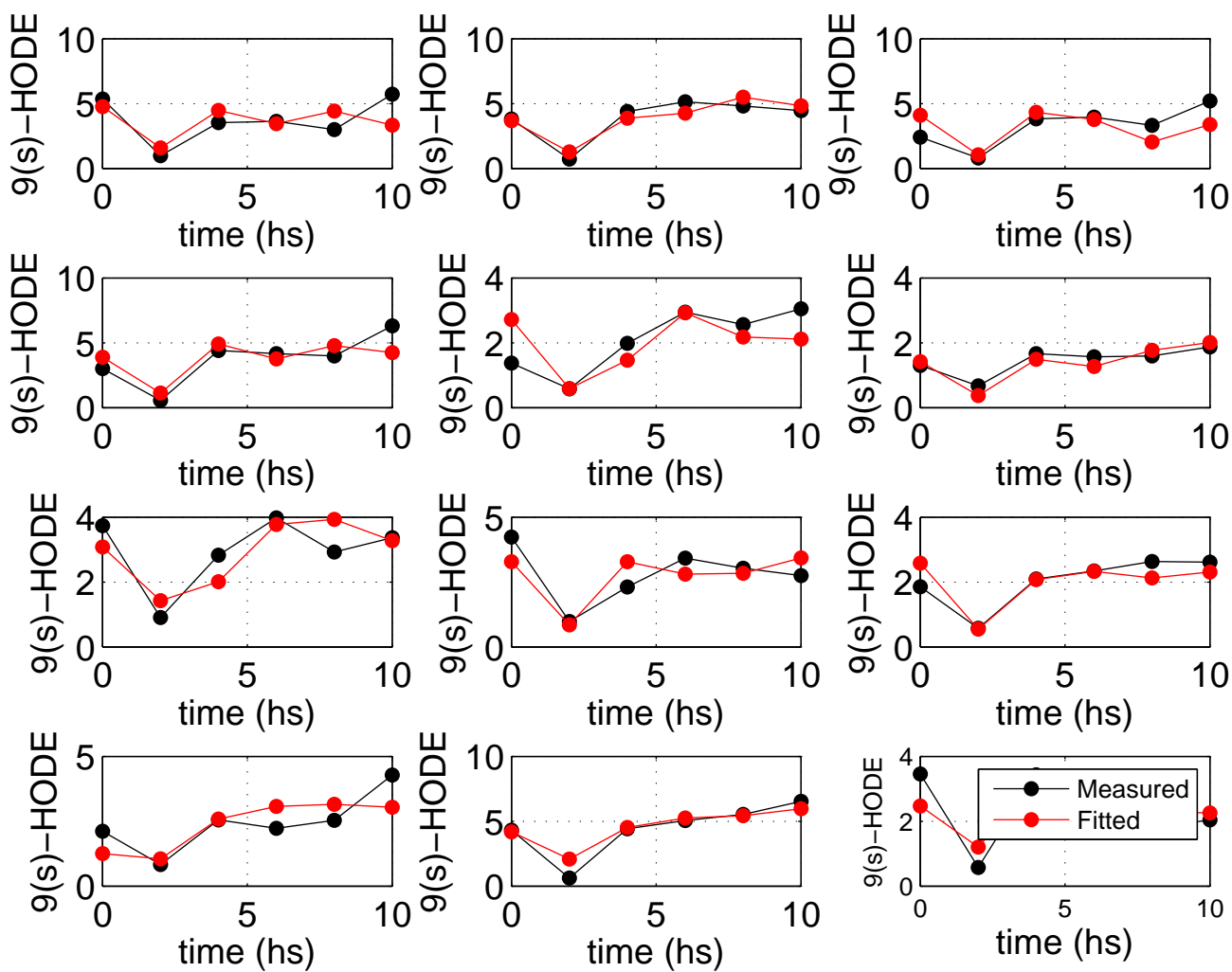

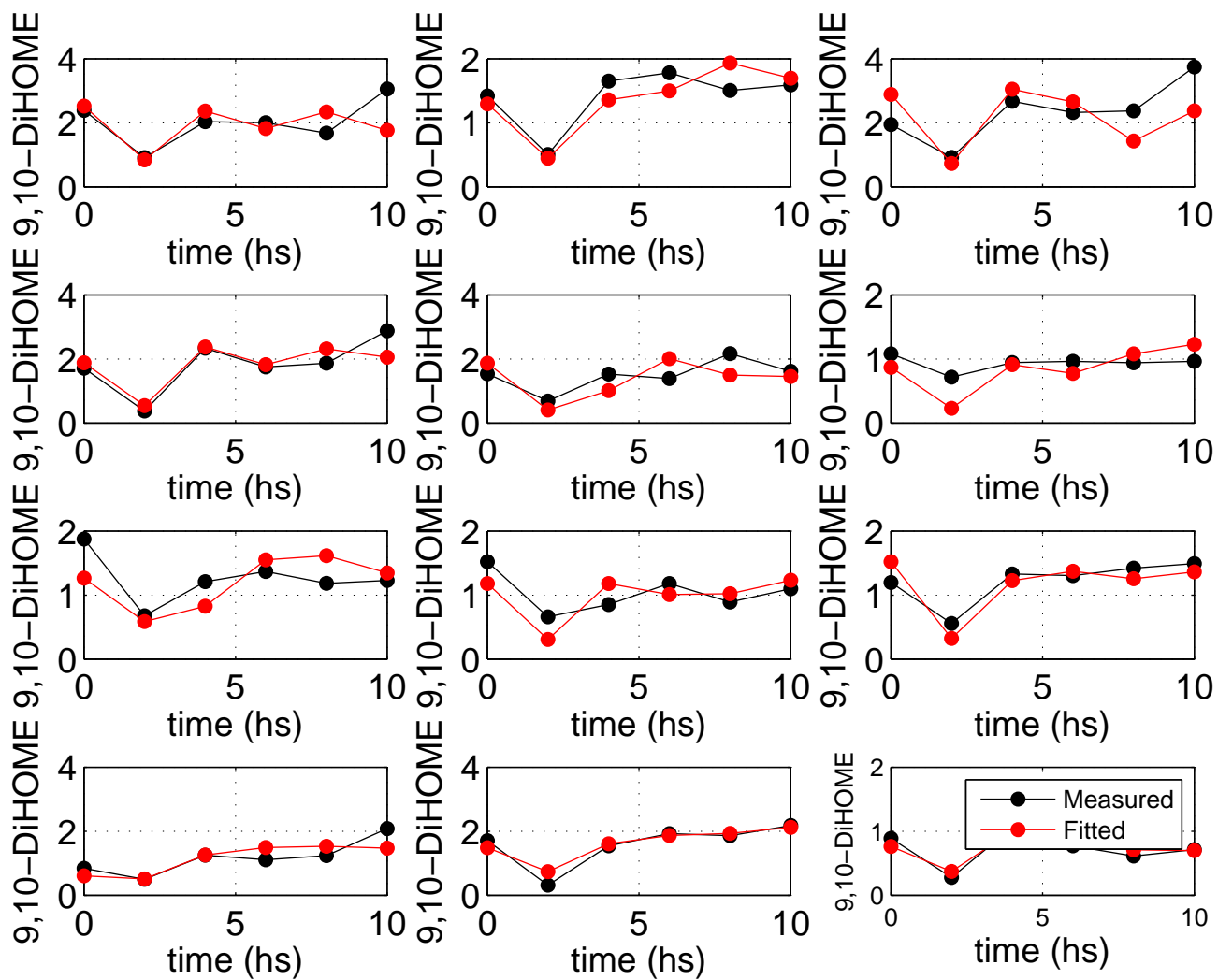

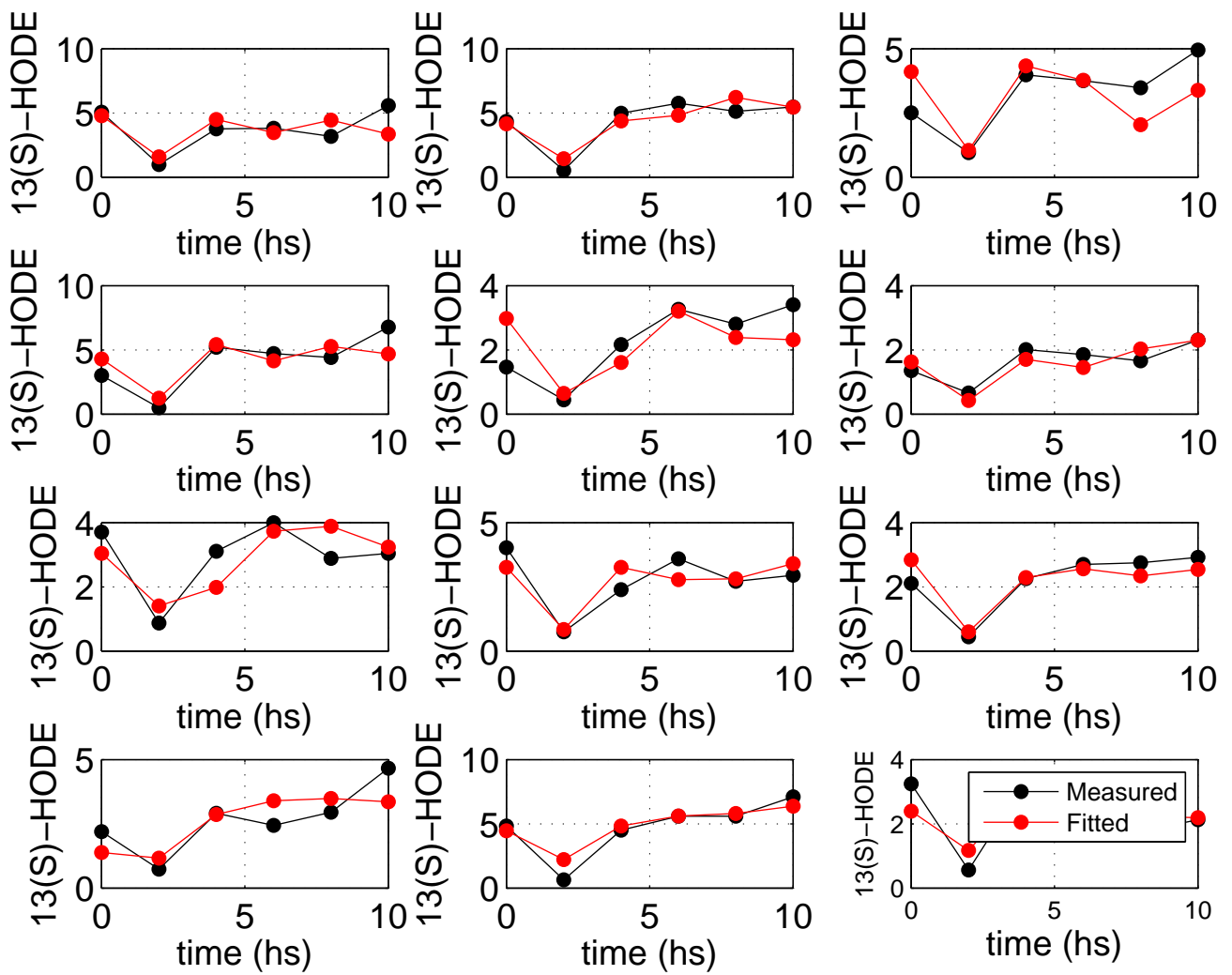

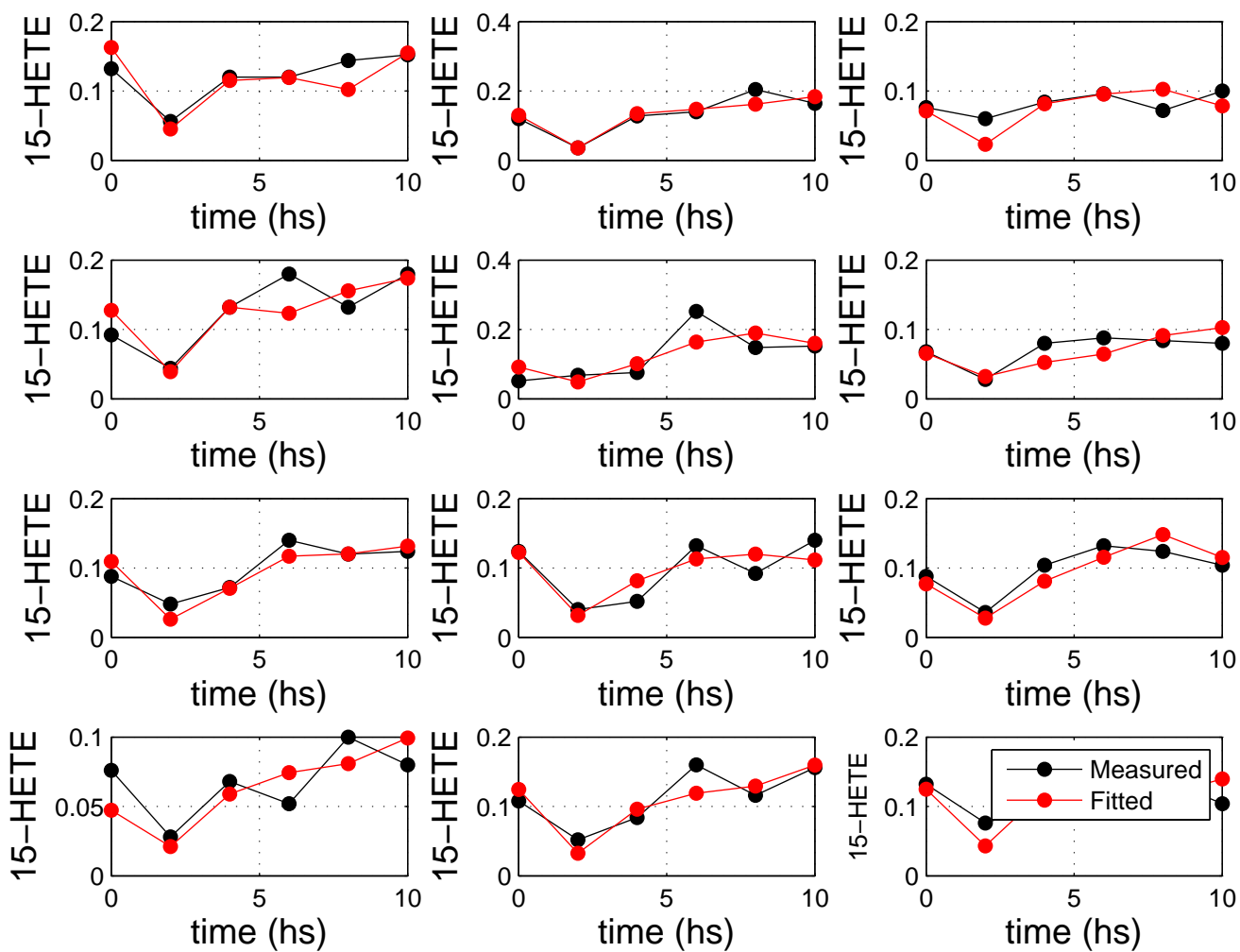

Supplement: S1 File — Plots of measured of and fitted concentration for AA and LA byproducts. (PDF) [file pone.0119856.s001.pdf]
